# Glycyrrhizin Reduces HMGB1 and Bacterial Load in *Pseudomonas aeruginosa* Keratitis

**DOI:** 10.1167/iovs.16-20103

**Published:** 2016-10

**Authors:** Sandamali A. Ekanayaka, Sharon A. McClellan, Ronald P. Barrett, Shikhil Kharotia, Linda D. Hazlett

**Affiliations:** Department of Anatomy & Cell Biology, Wayne State University School of Medicine, Detroit, Michigan, United States

**Keywords:** bacterial keratitis, mice, treatment

## Abstract

**Purpose:**

High mobility group box 1 (HMGB1) contributes to poor disease outcome in *Pseudomonas aeruginosa* keratitis. This study tests the prophylactic effect of treatment with HMGB1 inhibitors, glycyrrhizin (GLY) and its derivative, carbenoxolone (CBX), for Pseudomonas keratitis.

**Methods:**

We treated C57BL/6 (B6) mice subconjunctivally with GLY or CBX, infected with a noncytotoxic clinical isolate (KEI 1025) or a cytotoxic strain (ATCC 19660) of *P. aeruginosa*, and injected intraperitoneally with either agent. Clinical score, photography with a slit lamp, real-time RT-PCR, ELISA, myeloperoxidase (MPO) assay, bacterial plate count, histopathology, and absorbance assays were used to assess treatment efficacy and bacteriostatic activity.

**Results:**

After KEI 1025 infection, GLY treatment reduced HMGB1 (mRNA and protein levels) and improved disease outcome with significant reduction in mRNA levels of IL-1β, TLR4, CXCL2, and IL-12; protein expression (IL-1β, CXCL2); neutrophil infiltrate; and bacterial load. Treatment with GLY enhanced antimicrobial proteins, including CRAMP and mBD2, but not mBD3. Glycyrrhizin also reduced clinical scores and improved disease outcome in corneas infected with strain 19660. However, neither HMGB1 mRNA or protein levels were reduced, but rather, CXCL2 expression (mRNA and protein), neutrophil infiltrate, and bacterial load were reduced statistically. Treatment with GLY initiated 6 hours after infection reduced plate count; GLY also was bacteriostatic for KEI 1025 and ATCC 19660.

**Conclusions:**

Glycyrrhizin reduces HMGB1 and is protective against *P. aeruginosa*–induced keratitis with a clinical isolate that is noncytotoxic. It was similar, but less effective when used after infection with a cytotoxic strain, which did not reduce HMGB1.

Microbial keratitis is a sight threatening disorder associated with multiple risk factors, including use of extended wear soft contact lenses,^1^ ocular surface disease,^2^ ocular surgery,^3^ immunosuppression,^4,5^ and traumatic ocular surface accidents, particularly prevalent in developing countries.^6,7^ Among pathogens, the Gram-negative bacterium, *Pseudomonas aeruginosa*, remains a leading cause of contact lens–induced microbial keratitis,^8^ and infections result in ocular pain, stromal destruction, corneal thinning, and/or perforation, leading to vision loss, if untreated. Intensive antibiotic therapy is used to treat the disease, but with emerging antibiotic resistance of *P. aeruginosa* and other pathogens, it is advantageous to further test and develop potential alternative or adjunctive therapeutics.^9,10^

High mobility group box-1 (HMGB1), a member of the family of danger-associated molecular patterns (DAMPS) or alarmins, is a late mediator of inflammatory diseases.^11,12^ Extracellular HMGB1 binds to cell surface receptors such as the receptor for advanced glycation end products (RAGE) and Toll-like receptors (TLR) inducing release of proinflammatory molecules. This initiates an inflammatory cascade contributing to the pathogenesis of a variety of diseases, including ocular pathologies.^13,14^ Recently we described a strong correlation between HMGB1 and the severity of *P. aeruginosa* keratitis, which prompted further investigation of this inflammatory cytokine as a novel therapeutic target. In the study, in vivo assays using gene silencing and antibody neutralization of HMGB1 demonstrated an improvement in disease outcome.^15^

In this regard, it has been reported that glycyrrhizin (GLY) and one of its synthetic derivatives, carbenoxolone (CBX), bind HMGB1; and GLY has been shown to counteract its chemokine and cytokine mediated inflammatory cascade.^16–18^ Glycyrrhizin is a glycoconjugated triterpene extracted from licorice root (*Glycyrrhiza glabra*). It possesses numerous pharmacologic effects^19^ and has been shown effective in animal models of sepsis,^20^ colitis,^21^ lung,^22^ and brain^23^ injury. It is also used in clinical management to treat chronic hepatitis.^24^ Carbenoxolone is an anti-inflammatory drug prescribed for peptic ulcers.^25^ It also possesses protective effects in animal models for lung^26^ and cerebral ischemic injury.^27^ However, neither of these compounds has been tested in experimental keratitis induced by *P. aeruginosa*.

Thus, the objective of the current study was to determine which molecule, GLY or CBX, was most effective in treatment of Pseudomonas keratitis induced by a noncytotoxic clinical strain (KEI 1025). Evidence shows that GLY treatment optimally reduced HMGB1 expression (mRNA and protein) in KEI 1025–infected corneas and resulted in reduced corneal disease when compared with CBX. Decreased disease was consistent with reduction in IL-1β and CXCL2 expression (mRNA and protein), neutrophil infiltrate, bacterial plate count, and elevation of antimicrobial proteins. We also selectively tested the effect of GLY after infection with a cytotoxic strain (ATCC 19660) of the bacteria. Treatment with GLY also reduced corneal disease after infection with the cytotoxic strain. Reduced disease was consistent with decreased CXCL2 protein expression, neutrophil infiltrate, and bacterial plate count. However, neither HMGB1 mRNA nor protein levels were reduced compared with controls. Collectively, these data provide evidence that GLY reduces Pseudomonas keratitis whether induced by a noncytotoxic or cytotoxic strain, but is most effective against the noncytotoxic strain where HMGB1 is significantly reduced.

## Materials and Methods

### Mice

We purchased 8-week-old female C57BL/6 (B6) mice from the Jackson Laboratory (Bar Harbor, ME). Mice were housed in accordance with the National Institutes of Health guidelines. They were treated humanely and in compliance with the ARVO Statement for the Use of Animals in Ophthalmic and Vision Research.

### Bacterial Culture and Infection

We grew *P. aeruginosa* strains; KEI 1025, a noncytotoxic clinical isolate strain expressing *exoT* and *exoS*^28^ (Kresge Eye Institute, Detroit, MI, USA); and a cytotoxic strain, 19660 (American Type Culture Collection [ATCC] Manassas, VA, USA), which expresses *exoU* and *exoT*^29,30^ in peptone tryptic soy broth (PTSB) medium in a rotary shaker water bath at 37°C and 150*g* for 18 hours to an optical density (measured at 540 nm) between 1.3 and 1.8. Bacterial cultures were pelleted by centrifugation at 5500*g* for 10 minutes. Pellets were washed once with sterile saline, recentrifuged, resuspended, and diluted in sterile saline. Anesthetized (using anhydrous ethyl ether) mice were placed beneath a stereoscopic microscope at ×40 magnification. The left cornea was scarified with three 1-mm incisions using a sterile 25^5/8^ gauge needle. The wounded corneal surface was topically treated with 5 μL containing 1 × 10^7^ colony forming units (CFU)/μL (KEI 1025) or 1 × 10^6^ CFU/μL (ATCC strain 19660) of the *P. aeruginosa* suspension.

### Ocular Response to Bacterial Infection

An established corneal disease grading scale^31^ was used to assign a clinical score value to each infected eye at 1, 3, and 5 days postinfection (PI). Clinical scores were designated as: 0, clear or slight opacity, partially or fully covering the pupil; +1, slight opacity, fully covering the anterior segment; +2, dense opacity, partially or fully covering the pupil; +3, dense opacity, covering the entire anterior segment; and +4, corneal perforation or phthisis. Clinical scores were used to statistically compare disease severity and were accompanied by photographs using a slit lamp camera (5 days PI) to confirm and illustrate the response.

### GLY or CBX Treatment

The left eyes of B6 mice (*n* = 5/group/time) were injected with 5 μL of GLY 2 μg/μL (Sigma-Aldrich Corp., St. Louis, MO, USA) or PBS (control) subconjunctivally, 1 day before infection. Similar treatment was done using CBX (Sigma-Aldrich Corp.). All mice were injected intraperitoneally (IP) at 1 and 3 days PI with GLY 100 μL (2 μg/μL) or CBX (1 μg/μL) based on reports for drug concentrations of GLY or CBX used by other laboratories.^18,32^ We used PBS as control for all these experiments. In a separate similar experiment mice were treated 1 day before infection with GLY subconjunctivally and injected IP with GLY plus recombinant (r) HMGB1 (1 μg/100 μL) or GLY plus PBS. Intraperitoneal injections were similarly given at 1 and 3 days PI. In an additional experiment, KEI 1025–infected B6 mice were treated with 5 μL of 2 μg/μL GLY subconjunctivally at 6 hours PI and injected IP at 1 and 3 days PI with 100 μL of GLY (2 μg/μL).

### Real Time RT-PCR

Mice that were KEI 1025, infected with ATCC 19660, GLY- or PBS-treated were killed at 5 days PI and the normal, contralateral (uninfected), and infected cornea were harvested. For each cornea, total RNA was isolated (RNA STAT-60; Tel-Test, Friendswood, TX, USA) according to the manufacturer's instructions. Upon spectrophotometric quantification at 260 nm, 1 μg of each RNA sample was reverse transcribed using Moloney-murine leukemia virus (M-MLV) reverse transcriptase (Invitrogen, Carlsbad, CA, USA) to produce a cDNA template for the PCR reaction.^15^ Complementary (c)DNA products were diluted 1:25 with diethylpyrocarbonate (DEPC)-treated water. A 2-μL aliquot of diluted cDNA was used for the real-time RT-PCR reaction with real-time SYBR green/fluorescein PCR master mix (Bio-Rad Laboratories, Richmond, CA, USA) and primer concentrations of 10 μM (total 10 μL reaction volume). After a preprogrammed hot start cycle (3 minutes at 95°C), the parameters used for PCR amplification were: 15 seconds at 95°C and 60 sec at 60°C with the cycles repeated 45 times. Optimal conditions for PCR amplification of cDNA were established using routine methods.^33^ Levels of mRNA of HMGB1, RAGE, IL-1β, TLR2, TLR4, CXCL2, TNF-α, NLR family pyrin domain containing 3 (NLRP3), NLR family CARD domain containing 4 (NLRC4), IL-12, TGF-β, IL-10, single Ig IL-1-related receptor (SIGIRR) and interleukin 1 receptor-like 1 (ST2) were tested by real-time RT-PCR (CFX Connect real-time PCR detection system; Bio-Rad Laboratories). The fold differences in gene expression were calculated after normalization to β-actin and expressed as the relative mRNA concentration + SEM. The primer pair sequences used for real-time RT-PCR are shown in the Table.

**Table:**
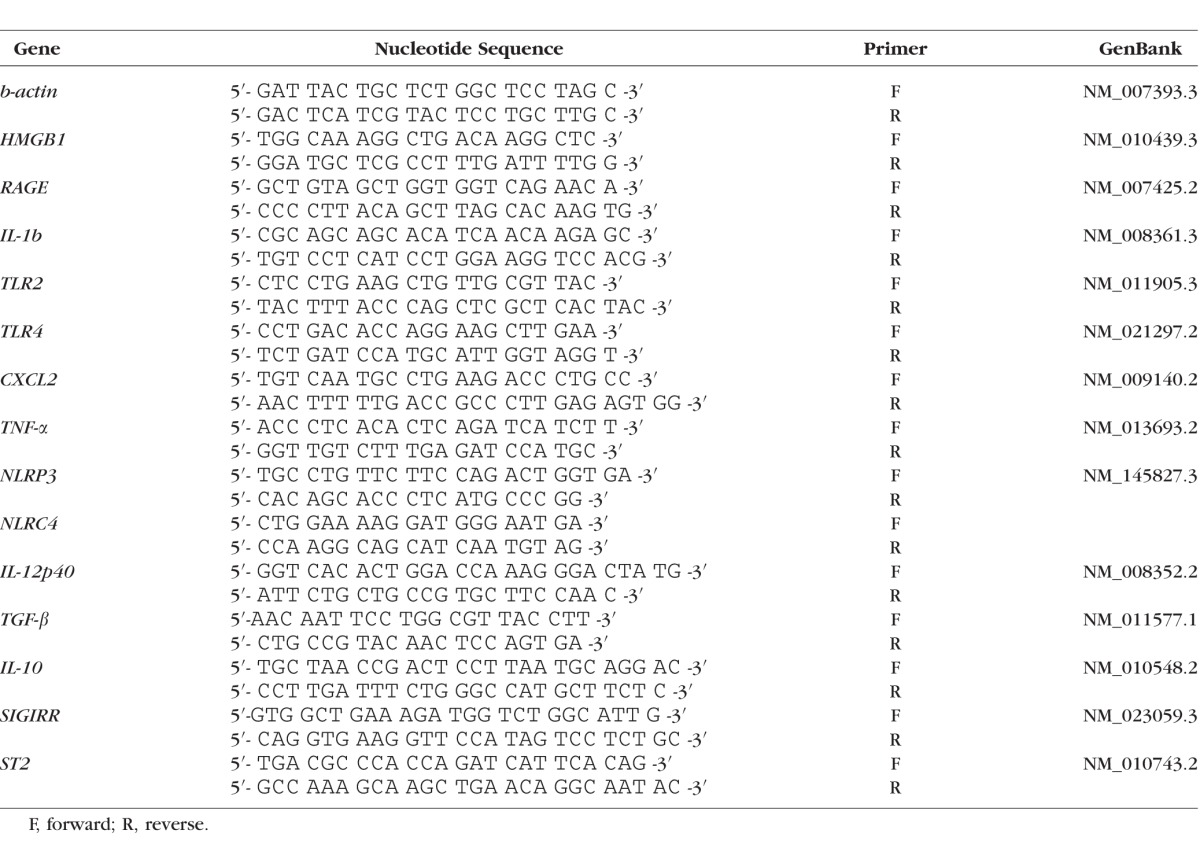
Nucleotide Sequence of the Specific Primers Used for PCR Amplification

### Enzyme-Linked Immunosorbent Assay

Mice that were KEI 1025, ATCC 19660–infected, GLY-/PBS-treated, or B6 (*n* = 5/group/time) were killed at 3 and 5 days PI and normal and infected corneas harvested. To quantify IL-1β and CXCL2 proteins, individual corneas were homogenized in 1 mL of 50 mM potassium phosphate buffer (pH 6.0) containing 0.5% hexadecyltrimethylammonium bromide (HTAB, Sigma-Aldrich Corp.). To quantify HMGB1, cathelicidin-related antimicrobial peptide (CRAMP), mouse beta defensin (mBD)2 and mBD3 protein levels, individual corneas were homogenized in 500 μL of PBS containing 0.1% Tween 20 (Sigma-Aldrich Corp.) and protease inhibitors (Roche, Indianapolis, IN, USA). Corneal homogenates were centrifuged at 12,000*g* for 10 minutes. A 50-μL aliquot of each supernatant was assayed in duplicate to quantify IL-1β, CXCL2, HMGB1, and mBD2 proteins. A 100-μL aliquot of CRAMP and mBD3 supernatant was assayed in duplicate to quantify corresponding protein levels. Undiluted supernatant aliquots were used to quantify all proteins except CXCL2 (1:2 dilution) and HMGB1 (1:5 dilution). An HMGB1 ELISA kit was purchased from Chondrex, Inc. (Redmond, WA, USA). We purchased CRAMP, mBD2 and mBD3 ELISA kits from MyBioSource, Inc. (San Diego, CA, USA). All other ELISA kits were purchased from R&D Systems (Minneapolis, MN, USA). Assays were run following the manufacturer's instructions. Sensitivities of the assays were: 2.31 pg/mL (IL-1β), 1.5 pg/mL (CXCL2), 0.8 ng/mL (HMGB1), 0.39 pg/mL (CRAMP), 2 pg/mL (mBD2), and 12 pg/mL (mBD3).

### Myeloperoxidase (MPO) Assay

This assay was used to quantitate neutrophils in the cornea of GLY- and PBS-treated mice (*n* = 5/group/time) infected with KEI 1025 or ATCC 19660. Individual corneas were removed at 3 and 5 days PI and homogenized in 1.0 mL of 50 mM phosphate buffer (pH 6.0) containing 0.5% HTAB. Samples were freeze-thawed four times and after centrifugation, 100 μL of the supernatant was added to 2.9 mL of 50 mM phosphate buffer containing *o*-dianisidine dihydrochloride (16.7 mg/mL, Sigma-Aldrich Corp.) and hydrogen peroxide (0.0005%). The change in absorbency was monitored at 460 nm for 5 minutes at 30-second intervals. The slope of the line was determined for each sample and used to calculate units of MPO/cornea. One unit of MPO activity is equivalent to ∼2 × 10^5^ neutrophils.^34^

### Quantification of Viable Bacteria

Mice were sacrificed at 3 and 5 days PI and KEI 1025 or ATCC 19660–infected corneas from GLY-, CBX- or PBS-treated (or GLY+/– rHMGB1) B6 mice were harvested (*n* = 5/group/time). Each cornea was homogenized in 1 mL of sterile saline (0.85% NaCl, pH 7.4) containing 0.25% BSA. A 100 μL of the corneal homogenate was serially diluted (1:10) in sterile saline containing 0.25% BSA. Selected dilutions were plated in triplicate on selective culture medium (Difco Pseudomonas Isolation Agar, BD Biosciences, Inc., Franklin Lakes, NJ, USA). Plates were incubated overnight at 37°C and viable bacteria manually counted. Results are reported as log_10_ CFU/cornea ± SEM.

### Histopathology

Whole eyes infected with KEI 1025 (*n* = 3/group/time) were enucleated from GLY- or PBS-treated B6 mice at 3 and 5 days PI. Eyes were immersed in PBS, rinsed, and fixed in 1% osmium tetroxide, 2.5% glutaraldehyde, and 0.2 M Sorenson's phosphate buffer (pH 7.4; 1:1:1) at 4°C for 3 hours. Eyes were rinsed with 0.1 M phosphate buffer, dehydrated in graded ethanols and propylene oxide, then infiltrated and embedded in Epon-araldite. Thick sections (1.5 μm) were cut, stained with Richardson's stain, observed, and photographed (Leica DM4000B, Leica Microsystems, Inc., Wetzlar, Germany), as described before.^35^

### Bacterial Growth and Minimum Inhibitory Concentration (MIC)

Bacterial cultures (KEI 1025 and ATCC 19660) were prepared as described above and bacterial growth examined as described before^36,37^ with the following modifications. Serial dilutions of GLY were prepared in PTSB (0–40 mg/mL) in sterile tubes and 5 mL of each bacterial culture (washed and reconstituted in saline) was added. The minimum inhibitory concentration of GLY was determined by spectrophotometric reading at 540 nm following incubation at 37°C for 18 hours. Values of MIC_50_ and MIC_90_ were defined as the lowest GLY concentrations that resulted in a 50% and 90% decrease in absorbance compared with the growth control, respectively.

### Statistical Analysis

The difference in clinical score between two groups was analyzed by the Mann-Whitney *U* test (GraphPad Prism, San Diego, CA, USA). For comparisons of three or more groups (RT-PCR), a 1-way ANOVA followed by the Bonferroni's multiple comparison test (GraphPad Prism) was used for analysis. For all other experiments in which two groups were compared (ELISA, MPO, plate count, RT-PCR, and MIC), an unpaired, 2-tailed Student's *t*-test was used to determine significance. For each test, *P* < 0.05 was considered significant and data are shown as mean + SEM. All experiments were repeated at least once to ensure reproducibility.

## Results

### Comparison of GLY and CBX Treatment After KEI 1025 Infection

**Figure 1:**
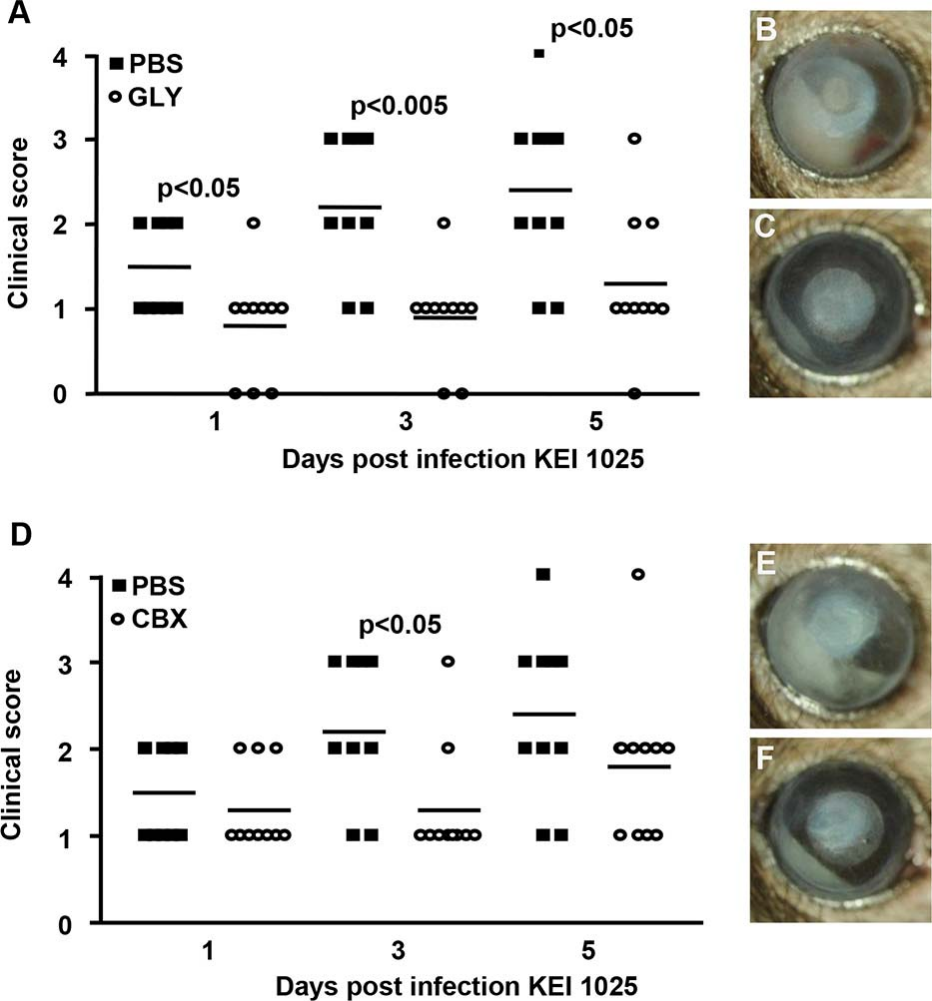
Disease response after infection with KEI 1025 and GLY or CBX treatment. Clinical scores (**A**) were reduced significantly at 1, 3, and 5 days PI in GLY- versus PBS-treated mice, but only at 3 days PI after CBX versus PBS treatment (**D**). Photographs taken with a slit lamp camera at 5 days PI from PBS (**B**) and GLY- (**C**) or PBS- (**E**) and CBX- (**F**) treated mice confirmed reduced opacity, but GLY-treated eyes appeared to have less infiltrate. Data were analyzed using a nonparametric Mann-Whitney *U* test. *Horizontal lines* indicate the median values. Magnification, ×8; *n* = 10/group/time.

Figure 1A shows that GLY-treated mice exhibited significantly less corneal disease with lower clinical scores than PBS-treated mice (*n* = 10 mice/group/time), at 1, 3, and 5 days PI (*P* < 0.05, *P* < 0.005, and *P* < 0.05). Photographs taken with a slit lamp of representative eyes from PBS- (Fig. 1B) and GLY- (Fig. 1C) treated mice at 5 days PI illustrate the reduced opacity evident in the GLY- versus PBS-treated group. For the CBX treatment group (Fig. 1D), statistically significant reduction in disease scores was seen at 3 (*P* < 0.05), but not at 5 (*P* = 0.2) days PI photographs taken with a slit lamp of representative eyes from PBS- and CBX-treated groups (Figs. 1E, 1F, respectively) are shown at 5 days PI.

### Real-Time RT-PCR: GLY/CBX Treatment After KEI 1025 Infection

**Figure 2:**
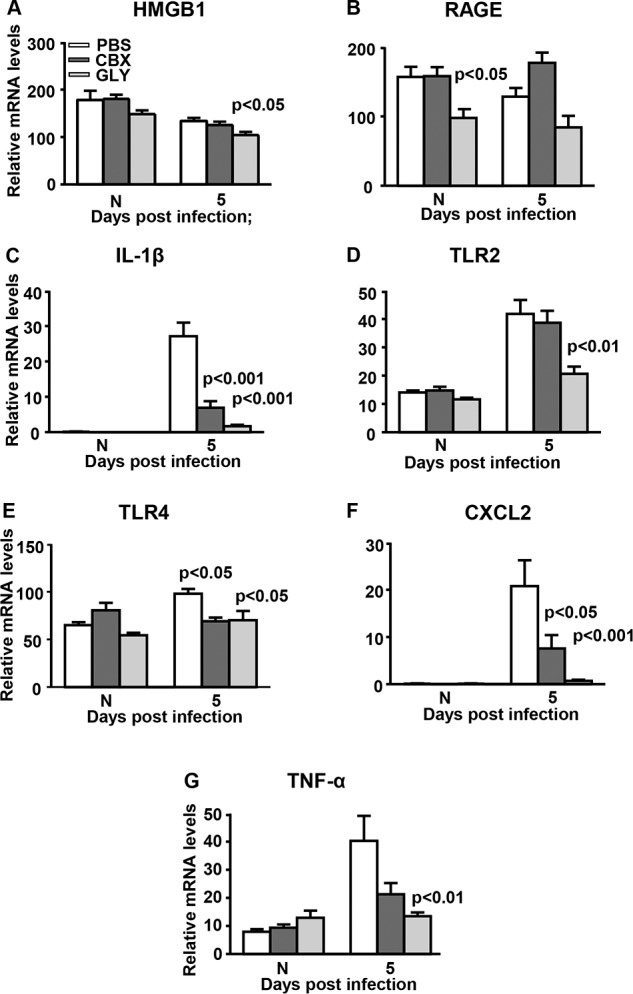
Real-time RT-PCR after infection with KEI 1025. (**A**–**G**) At 5 days PI, corneal mRNA levels of HMGB1 (**A**), TLR2 (**D**), and TNF-α (**G**) were reduced significantly only in GLY-treated corneas; RAGE (**B**) was not different statistically between groups; IL-1β (**C**), TLR4 (**E**), and CXCL2 (**F**) were reduced significantly after GLY and CBX versus PBS treatment. No difference in mRNA levels was seen for normal uninfected (N) cornea except the significant reduction for RAGE (**B**) in GLY-treated mice. Data are mean + SEM analyzed using 1-way ANOVA followed by the Bonferroni's multiple comparison test. *n* = 10/group/time.

**Figure 3:**
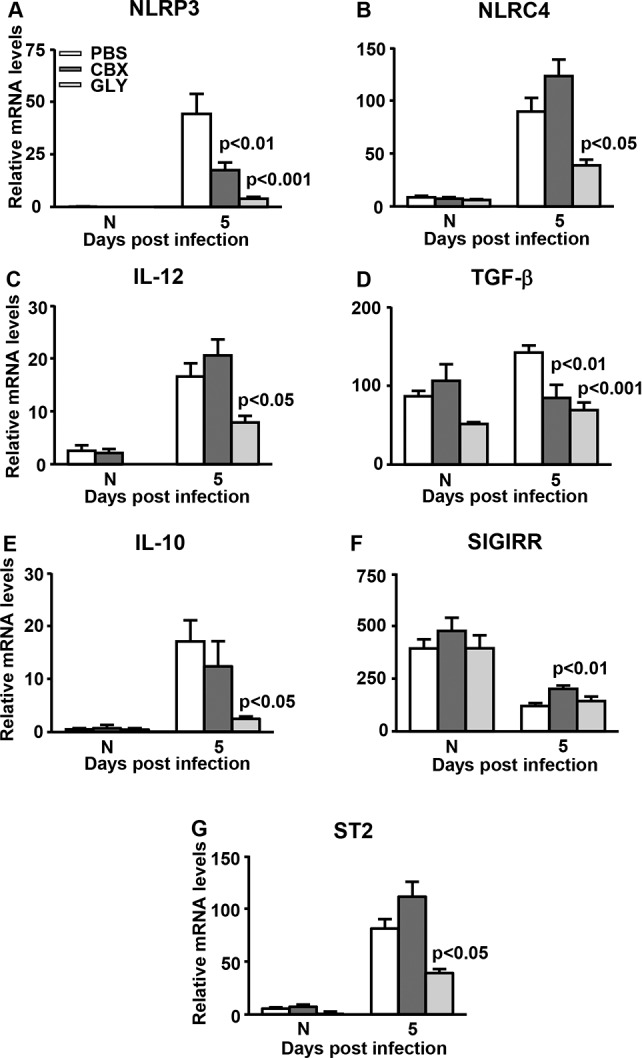
Real-time RT-PCR after infection with KEI 1025. At 5 days PI, corneal mRNA levels of: NLRP3 (**A**) and TGF-β (**D**) were reduced significantly after GLY or CBX treatment; NLRC4 (**B**), IL-12 (**C**), IL-10 (**E**), and ST2 (**G**) were reduced significantly only after GLY treatment; SIGIRR (**F**) was increased significantly only with CBX treatment. No difference between groups was seen for normal uninfected cornea. All data are mean + SEM and were analyzed using 1-way ANOVA followed by the Bonferroni's multiple comparison test. *n* = 10/group/time.

Focusing on 5 days PI, GLY significantly reduced HMGB1 (*P* < 0.05), while no difference was seen for CBX when compared with PBS treatment (Fig. 2A). Treatment with GLY-reduced RAGE mRNA levels (Fig. 2B) when compared with PBS but not significantly. Levels of IL-1β mRNA were significantly reduced by GLY (*P* < 0.001) and by CBX (*P* < 0.001) when compared with PBS (Fig. 2C). We found GLY significantly reduced TLR2 (*P* < 0.01), while CBX treatment did not differ from PBS controls (Fig. 2D). Toll-like receptor 4 mRNA was reduced by GLY (*P* < 0.05) and CBX (*P* < 0.05) similarly when compared with PBS (Fig. 2E). Protein CXCL2 was reduced by GLY (*P* < 0.001) and by CBX (*P* < 0.05) when compared with PBS (Fig. 2F). Levels of TNF-α mRNA were reduced by both GLY and CBX, but only GLY (*P* < 0.01) was significant compared with control levels (Fig. 2G). Glycyrrhizin and CBX also reduced mRNA levels for NLRP3 (*P* < 0.001 and *P* < 0.01), and for TGF-β (*P* < 0.001 and *P* < 0.01; Figs. 3A, 3D). However, only GLY reduced mRNA levels of NLRC4 (*P* < 0.05) and IL-12 (*P* < 0.05) compared with CBX or PBS treatment (Figs. 3B, 3C). GLY also reduced anti-inflammatory molecules IL-10 (*P* < 0.05, Fig. 3E), and ST2 (*P* < 0.05, Fig. 3G), while CBX increased modestly SIGIRR mRNA levels (*P* < 0.01, Fig. 3F). For all normal corneas, GLY significantly reduced mRNA levels only for RAGE (Fig. 2B, *P* < 0.05), with no differences for the other molecules tested among the three groups (Figs. 2, 3).

### ELISA, MPO, Histopathology and Plate Count

**Figure 4:**
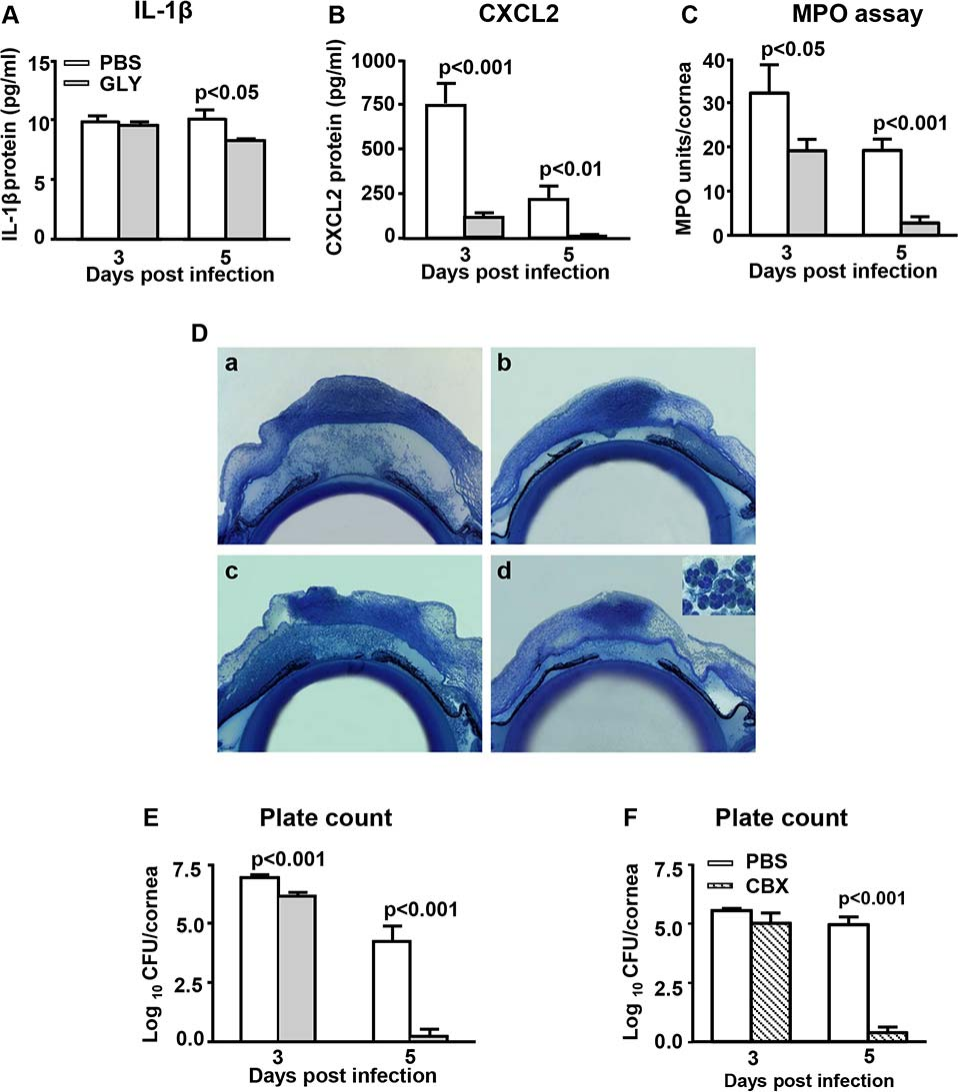
Histopathology, ELISA, MPO assay, and plate count after KEI 1025 infection. Corneal protein levels of IL-1β (**A**) were reduced significantly at 5 days PI with no difference at 3 days PI after GLY treatment. CXCL2 (**B**) protein levels were reduced significantly at 3 and 5 days PI in GLY versus PBS-treated cornea. Levels of MPO (**C**) and viable bacterial plate count was reduced significantly at 3 and 5 days PI in GLY versus PBS-treated cornea (**E**), but only at 5 days PI in CBX versus PBS-treated cornea (**F**). All data are mean + SEM and were analyzed using a 2-tailed Student's *t*-test (*n* = 5/group/time). Histopathology (**D**) revealed a heavy cellular infiltrate in the stroma and anterior chamber of the PBS-treated eyes at 3 and 5 days PI (**D**[**a**], **D**[**c**], respectively). The eyes of GLY-treated mice showed fewer infiltrated cells into cornea and anterior chamber, and less edema, at 3 and 5 days PI (**D**[**b**], **D**[**d**], respectively). Inset shows a predominant neutrophil infiltrate which is greatly reduced in the GLY-treated mice. Magnification, ×30; inset, ×490, *n* = 3/group/time.

Glycyrrhizin treatment significantly decreased mRNA expression of HMGB1, and thus, its effects were studied further for effects on chemotactic cytokines, and neutrophilic infiltrate. For plate counts, we comparatively tested GLY and CBX. Treatment with GLY significantly decreased IL-1β protein at 5 days PI (Fig. 4A, *P* < 0.05), but was no different from control values at 3 days PI CXCL2 protein expression was significantly decreased both at 3 (*P* < 0.001) and 5 (*P* < 0.01) days PI (Fig. 4B). Consistent with reduced levels of these neutrophil chemoattractant cytokines, an MPO assay (Fig. 4C) revealed decreased neutrophils in cornea at both 3 and 5 days PI (*P* < 0.05 and *P* < 0.001, respectively; Fig. 4C). The eyes that were KEI 1025 infected and treated with GLY or PBS also were examined histopathologically (Fig 4D). Reflective of the MPO data, GLY-treated eyes showed a markedly reduced cellular infiltrate (predominantly PMN, as illustrated in the inset in Figure 4D[d]) in the corneal stroma and anterior chamber both at 3 and 5 days PI (Figs. 4D[b] and 4D[d], respectively). In contrast, the PBS-treated eyes showed a heavier cellular infiltrate in the corneal stroma, denudation of the corneal epithelium, stromal degradation and edema both at 3 and 5 days PI (Figs. 4D[a] and 4D[c], respectively). The bacterial load in the corneas of GLY-treated mice was reduced at both 3 and 5 days PI (*P* < 0.001 and *P* < 0.001, respectively; Fig. 4E). After treatment with CBX, plate count was significantly reduced (*P* < 0.001) at 5 days PI (Fig. 4F), but not at 3 days PI as seen with GLY treatment.

### GLY Reduces HMGB1 and Enhances Antimicrobial Peptides

**Figure 5:**
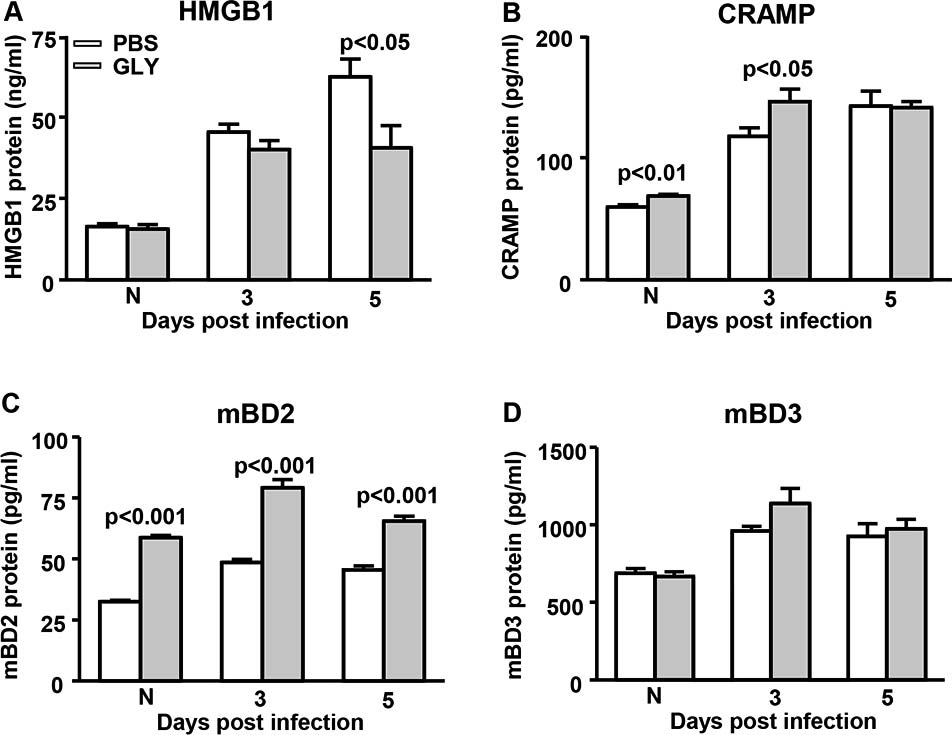
Levels of HMGB1, CRAMP, mBD2, and mBD3 ELISA after infection with KEI 1025. After GLY versus PBS treatment, protein levels of HMGB1 (**A**) were reduced significantly at 5 days PI, but not at 3 days PI or in the N cornea. Treatment with GLY versus PBS significantly upregulated CRAMP (**B**) protein expression in the normal uninfected cornea and at 3 days PI, but not at 5 days PI. Protein levels of mBD2 (**C**) were increased significantly in normal uninfected, and 3 and 5 days PI corneas in GLY- vs PBS-treated mice. Protein levels of mBD3 (**D**) were not significantly different after GLY treatment in normal uninfected cornea, and 3 or 5 days PI. All data are mean + SEM and were analyzed using a 2-tailed Student's *t*-test (*n* = 5/group/time).

After infection with KEI 1025, GLY treatment reduced HMGB1 protein significantly at 5 (*P* < 0.05), but not 3 days PI and did not differ in the normal cornea between groups (Fig. 5A). Treatment affected expression of antimicrobial peptides and upregulated the expression of CRAMP significantly in the normal, uninfected cornea (*P* < 0.01) and at 3 days PI (*P* < 0.05) with no difference between groups observed at 5 days PI (Fig. 5 B). Protein mBD2 (Fig. 5C) was significantly upregulated at 3 and 5 days PI (*P* < 0.001 and *P* < 0.001, respectively) and also was higher in the normal, contralateral uninfected eye (*P* < 0.001). Treatment with GLY versus PBS did not differ significantly at 3 or 5 days PI or in the normal cornea for mBD3 protein (Fig. 5D).

### GLY+/– rHMGB1

**Figure 6:**
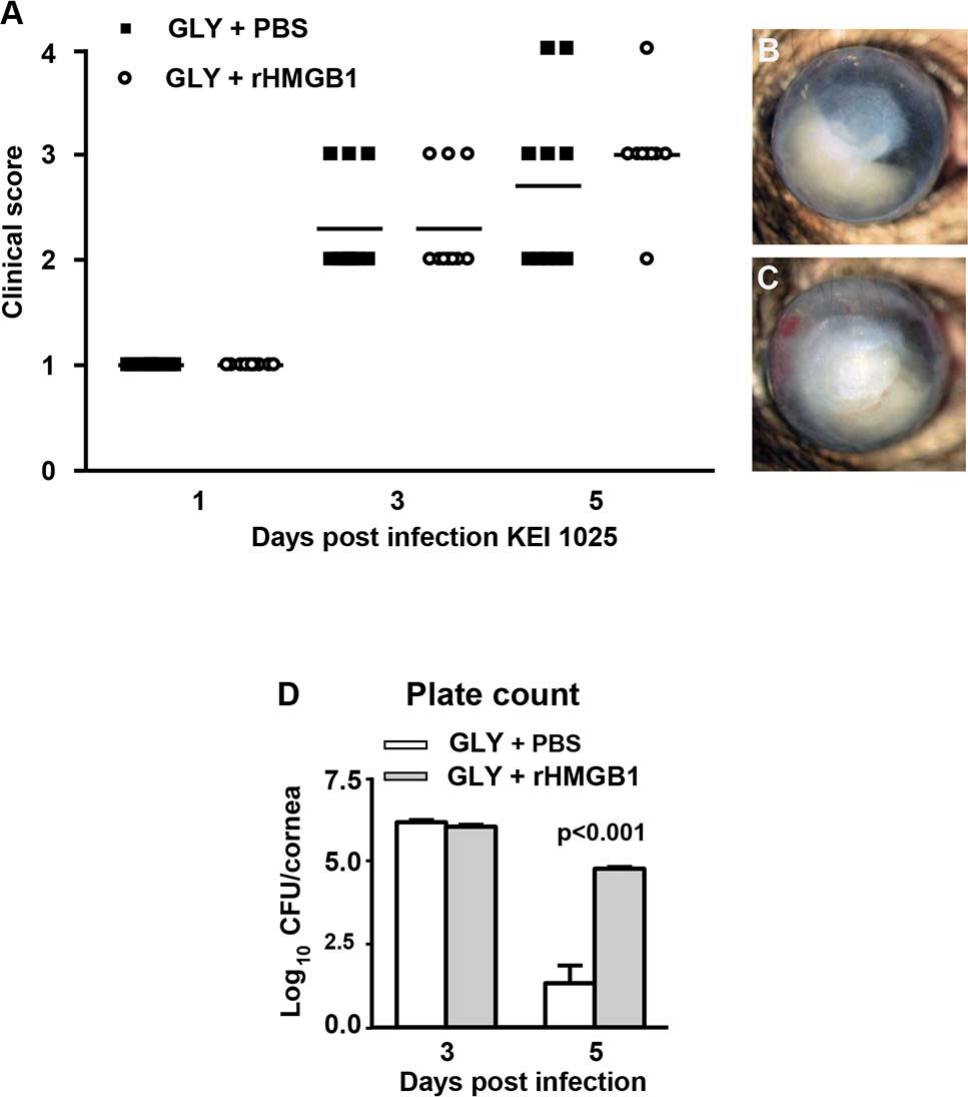
Treatment with GLY+/– rHMGB1 and KEI 1025 infection. Clinical scores (**A**) were not significantly different between treatment groups at all times tested. Photographs taken with a slit lamp camera at 5 days PI from (**B**) GLY- and (**C**) GLY+ rHMGB1–treated mice showed slightly increased opacity in the cornea of the latter-treated mice. Plate counts (**D**) showed no differences between groups at 3 days PI, but a significant increase in bacterial load at 5 days PI after GLY + rHMGB1 treatment. Clinical score data were analyzed using a nonparametric Mann-Whitney *U*-test. *Horizontal lines* indicate the median values. Magnification, ×8; *n* = 10/group/time. Plate count data are shown as mean + SEM and were analyzed using a 2-tailed Student's *t*-test (*n* = 5/group/time).

To further test whether GLY reduction of HMGB1 levels was important for disease outcome or that it mainly reduced bacterial plate count which then downregulated the inflammatory response, enhancing antimicrobial peptides, and improving disease, we treated mice infected with KEI 1025 using both GLY and rHMGB1. Clinical scores (Fig. 6A) showed no significant differences between treatment groups at all times tested; however, slit lamp photographs showed enhanced opacity in rHMGB1- and GLY-treated (Fig. 6C) versus GLY- and PBS-treated (Fig. 6B) corneas. Adding rHMGB1 to GLY treatment also significantly (*P* < 0.001) enhanced bacterial plate count at 5 days PI, but no difference was seen between groups at 3 days PI (Fig. 6D).

### GLY Treatment Using Strain 19660

**Figure 7:**
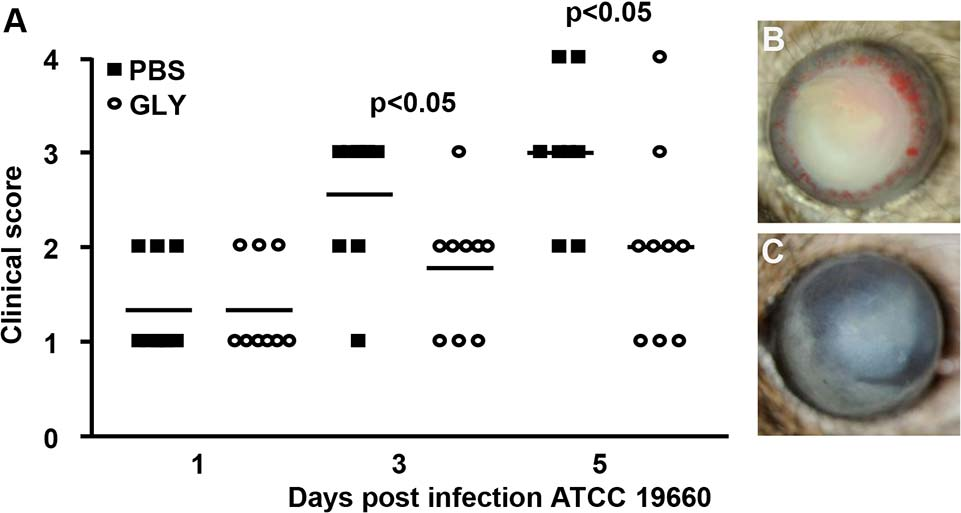
Treatment with GLY after infection with strain 19660. Clinical scores (**A**) were reduced significantly at 3 and 5 days PI, but not at 1 day PI in GLY versus PBS-treated mice (*n* = 9/group/time). Photographs taken with a slit lamp at 5 days PI from (**B**) PBS- and (**C**) GLY-treated mice confirmed reduced opacity after GLY treatment. *Horizontal lines* indicate the median values. Data were analyzed using a nonparametric Mann-Whitney *U*-test. Magnification, ×8.

Severity of disease was graded (clinical scores) and photographs taken with a slit lamp camera following infection with cytotoxic ATCC strain 19660. Treatment with GLY versus PBS (Fig. 7A) showed reduced clinical scores that were significant both at 3 (*P* < 0.05) and 5 days PI (*P* < 0.05), but not at 1 day PI. Photographs taken with a slit lamp camera confirmed less disease in the GLY- (Fig. 7C) compared with the PBS-treated eye (Fig. 7B).

### RT-PCR, ELISA, MPO Assay and Plate Count for ATCC Strain 19660

**Figure 8:**
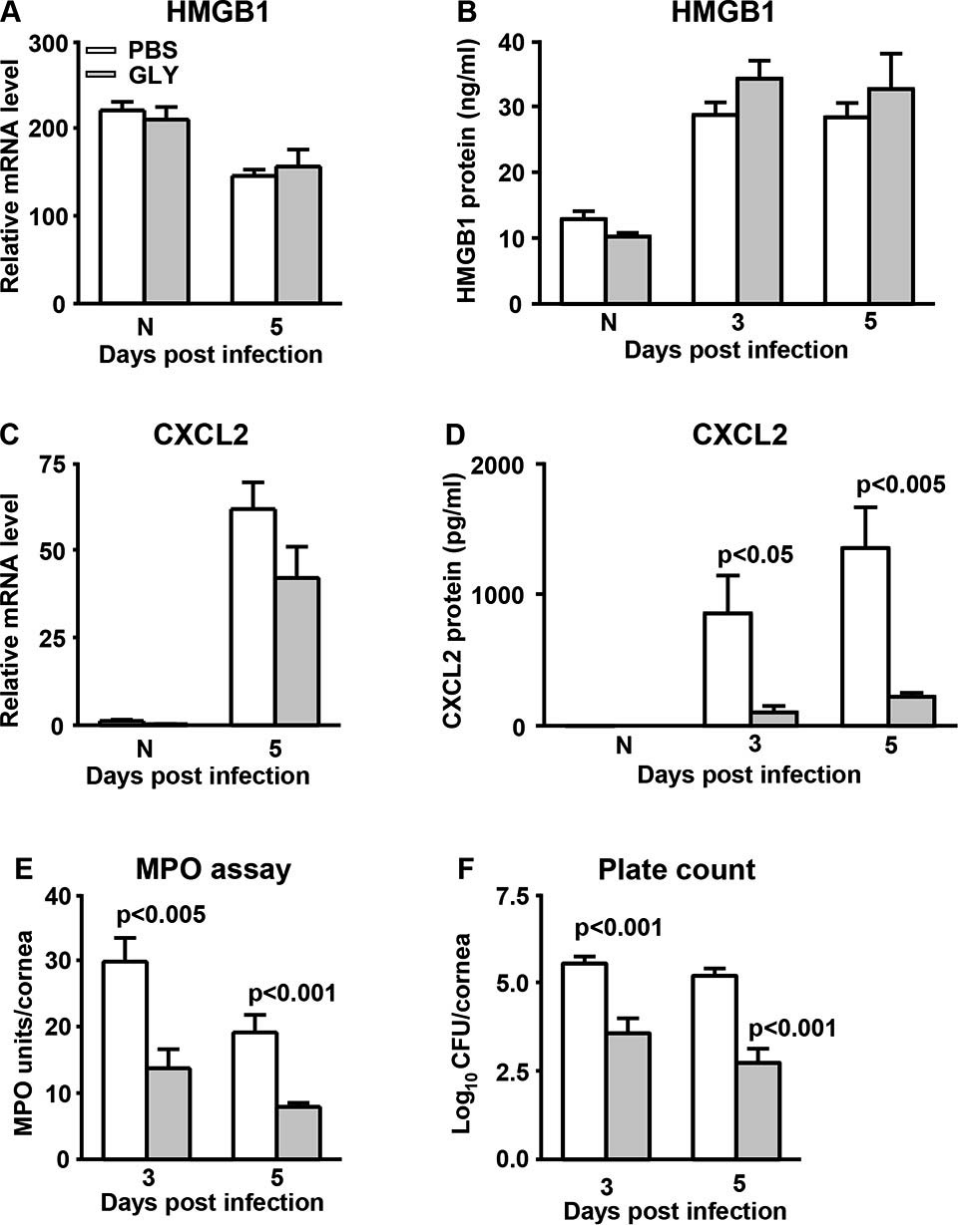
Real-time RT-PCR, ELISA, MPO assay, and plate count after infection with strain 19660. (**A**) Extracellular HMGB1 mRNA and (**B**) protein levels were not different significantly after GLY treatment for normal uninfected or infected corneas compared with PBS control. Treatment with GLY versus PBS slightly reduced corneal mRNA levels of CXCL2 (**C**) at 5 days PI CXCL2 protein levels (**D**) were reduced significantly at 3 and 5 days PI after GLY versus PBS treatment. Levels of MPO (**E**) and viable bacterial plate count (**F**) were reduced significantly at 3 and 5 days PI in GLY versus PBS-treated corneas. All data are mean + SEM and were analyzed using a 2-tailed Student's *t*-test (*n* = 5/group/time).

Relative mRNA levels for HMGB1 (Fig. 8A) were no different for both the uninfected, normal cornea, and GLY-treated infected cornea at 5 days PI. Protein analysis confirmed the mRNA data, with no significant differences between the normal cornea or the two infected groups (Fig. 8B). Levels of mRNA of CXCL2 (Fig. 8C) were slightly, but not significantly decreased at 5 days PI after GLY versus PBS treatment. Protein levels (Fig. 8D) were downregulated significantly at 3 and 5 days PI (*P* < 0.05 and *P* < 0.005, respectively) in GLY- versus PBS-treated mice. No differences in the normal cornea for mRNA or protein were detected between groups. An assay with MPO (Fig. 8E) revealed a significant decrease in neutrophils in the cornea at 3 and 5 days PI (*P* < 0.005 and *P* < 0.001, respectively) after GLY versus PBS treatment. Bacterial load (Fig. 8F) was significantly reduced in the corneas of GLY versus PBS-treated mice at both 3 and 5 days PI (*P* < 0.001 and *P* < 0.001, respectively).

### GLY Posttreatment and Plate Count, KEI 1025

**Figure 9:**
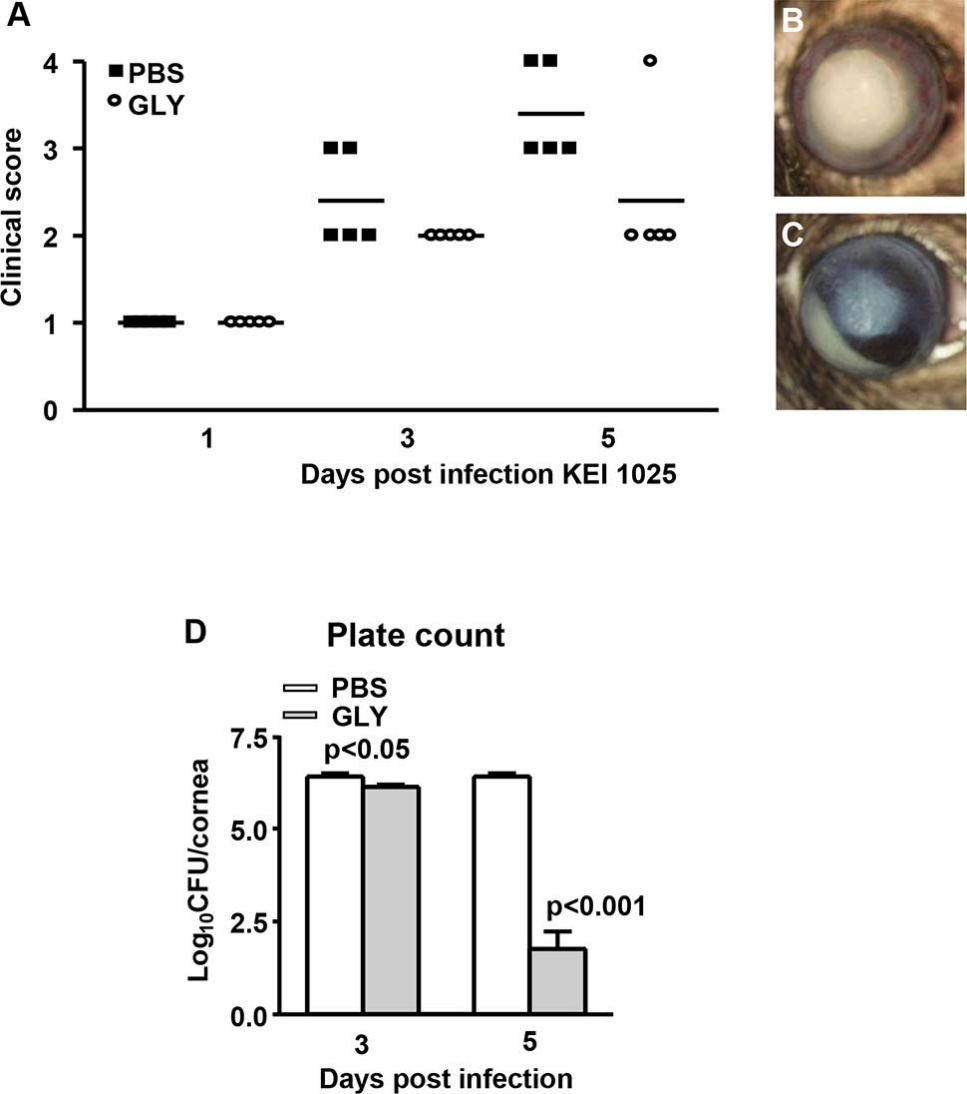
Posttreatment with GLY and bacterial plate count after KEI 1025 infection. Clinical scores (**A**) were reduced at 3 and 5 days PI but not at 1 day PI in GLY versus PBS-treated mice (*n* = 5/group/time). Horizontal lines indicate the median values. Photographs taken with a slit lamp at 5 days PI from (**B**) PBS and (**C**) GLY-treated mice illustrate reduction in opacity after GLY treatment. Data were analyzed using a nonparametric Mann-Whitney *U* test. Magnification, ×8. Viable bacterial plate count (**D**) was reduced significantly at 3 and 5 days PI after GLY versus PBS treatment. All data are mean + SEM and were analyzed using a 2-tailed Student's *t*-test (*n* = 5/group/time).

Ocular response to infection was graded **(**clinical scores) and photographs were taken with a slit lamp camera of the B6 mice infected with KEI 1025 and treated subconjunctivally (6 hours PI) and injected intraperitoneally (1 and 3 days PI) with GLY. Reduced clinical scores were seen only at 3 and 5 days PI after GLY versus PBS treatment (Fig. 9A). Photographs taken with a slit lamp camera documented decreased opacity in the GLY (Fig. 9C) compared with the PBS-treated eye (Fig. 9B). Nonetheless, despite the clinical appearance, bacterial load (Fig. 9D) was reduced significantly at both 3 and 5 days PI (*P* < 0.05 and *P* < 0.001, respectively) after GLY treatment.

### Bacterial Growth and MIC

**Figure 10:**
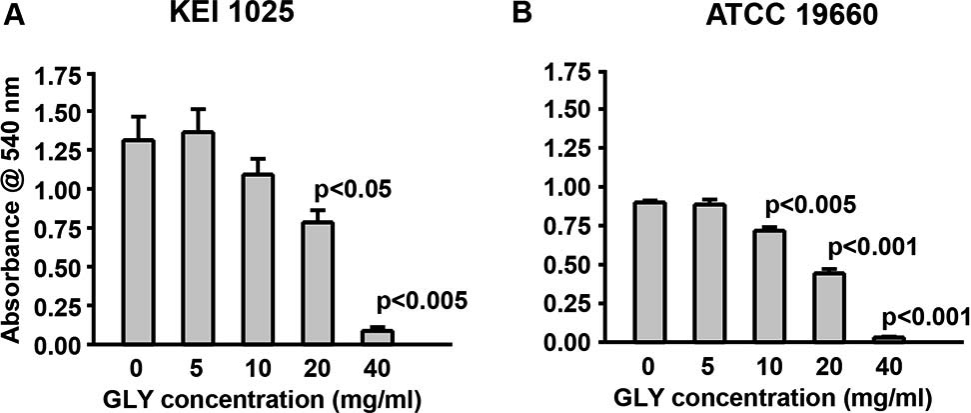
Effect of GLY on *P. aeruginosa* growth. Treatment with GLY significantly reduced absorbance values for strain KEI 1025 (**A**) at 20 and 40 mg/mL, but not at other concentrations. For strain ATCC 19660 (**B**) absorbance values were significantly reduced for cultures grown with 10, 20 or 40 mg/mL, but not with GLY 5 mg/mL. All data are mean + SEM and were analyzed using a 2-tailed Student's *t*-test (*n* = 3/group/strain).

For strain KEI 1025 (Fig. 10A), absorbance values were significantly reduced only for bacterial cultures grown with 20 (*P* < 0.05) or 40 mg/mL (*P* < 0.005) GLY. For strain 19660 (Fig. 10B), absorbance values were significantly reduced for bacterial cultures grown with 10 (*P* < 0.01), 20 (*P* < 0.001) or 40 mg/mL (*P* < 0.001) of GLY. For both bacterial strains, MIC_50_ and MIC_90_ values of GLY were 20 and 40 mg/mL, respectively.

## Discussion

Emerging antibiotic resistance is a major concern when treating microbial infections, and development of alternative therapies remains an urgent need.^9,10^ In this regard, extracellular HMGB1, a member of a family of molecules referred to as DAMPS or alarmins, contributes to the pathogenesis of Pseudomonas keratitis,^15^ as well as other infectious^13,38^ and noninfectious diseases.^38,39^ Extracellular HMGB1 is a late mediator of the inflammatory response,^12^ in contrast to other proinflammatory molecules^12^ (e.g., TNF-α, IL-1β, and IFN-α) that are released by activated immune cells early in the disease response. For example, in sepsis and endotoxemia, HMGB1 levels plateaued between 24 to 36 hours after infection. This wide therapeutic window^12^ and the strong correlation between HMGB1 and the pathogenesis of various infectious diseases^13^ suggest that it may provide an optimum target for treatment and clinical use. In fact, previous preclinical animal studies have reported various strategies to do precisely that. Unfortunately, many of those approaches have drawbacks that limit their use in a clinical setting.^38^ Case in point, recently, this laboratory demonstrated the use of a small interfering RNA (siHMGB1) to knock down HMGB1 in a mouse model of *P. aeruginosa* keratitis.^15^ Treatment with siHMGB1 led to improved disease outcomes along with reduction in proinflammatory cytokines, an increase in anti-inflammatory cytokines and reduced neutrophil infiltration. For the current study, we used a higher bacterial concentration for the clinical isolate than was used in the above paper^15^ where 1 × 10^6^ CFU/μL was used for both the cytotoxic and the clinical isolate strains and silencing (scrambled treatment for controls siRNA for HMGB1) was our experimental approach to decrease HMGB1 levels. This proof of principle study established HMGB1 as a target for treatment. In the GLY studies reported herein, we used 1 × 10^6^ CFU/μL of the clinical isolate in two separate preliminary experiments (data not shown) and found no significant difference between the PBS control (light opacity/infection) and GLY-treated mice. This led us to test an inoculum of 1 × 10^7^ CFU/μL, which provided consistent statistically significant data for the clinical isolate with GLY treatment compared with PBS controls as shown in the manuscript.

Silencing or knockdown in a clinical setting also would be fraught with potential issues such as specificity and longevity of treatment and possible toxicity problems. Furthermore, HMGB1 has numerous other cellular functions such as regulation of genomic stability and autophagy,^40^ as well as cell differentiation and apoptosis.^41^

In this regard, GLY and its derivative CBX, small triterpenoid saponin molecules, directly bind to HMGB1 without interfering with its secondary structure and GLY inhibits extracellular HMGB1-mediated mitogenic and chemotactic functions.^17^ Preclinical animal studies of inflammatory diseases such as sepsis revealed that both GLY and CBX inhibit expression, extracellular secretion and cytokine activity of HMGB1 and other proinflammatory cytokines.^16,38^ However, the underlying mechanisms of GLY or CBX regulation of the extracellular cytokine activity of HMGB1 in inflammatory diseases remained poorly understood. Recent studies, however, using an endotoxemic mouse model and LPS-treated RAW 264.7 cells shed some light on this issue, showing that GLY treatment induced heme oxygenase 1 expression via a p38MAPK/Nrf2 pathway, inhibiting HMGB1 extracellular secretion and hence its extracellular cytokine activity.^42^ In addition, GLY has been used clinically to treat chronic hepatitis,^24^ allergic conjunctivitis, and blepharitis,^14^ while CBX was used to treat esophageal ulceration^25^ with no signs of adverse events or drug toxicity in patients for either agent.

The study reported herein, examining the effects of GLY versus CBX treatment, provides the first evidence that GLY treatment improves disease outcome of Pseudomonas keratitis induced by a noncytotoxic clinical strain KEI 1025. When comparing the two agents, CBX did not reduce mRNA expression of HMGB1 significantly, optimally reduce several proinflammatory molecules (IL-1β, TLR2, CXCL2, TNF-α, IL-12, TGF-β) or inflammasome expression (NLRP3 or NLRC4) nor optimally improve disease outcome compared with GLY. The two agents also were not similar kinetically in reducing bacterial load as GLY treatment was effective at both 3 and 5 days PI, whereas CBX reduced load significantly only at 5 days PI. In this regard, Dembinska-Kiec et al.^43^ have shown that CBX treatment increased prostaglandin levels and the effects of the nitric oxide (NO) system in ethanol induced injury in the rat gastric mucosa. Hypothetically, although not tested in our model, CBX could have elevated NO levels in the infected cornea, which would contribute to bacterial killing. The latter hypothesis is consistent with past work from this laboratory that provided evidence that NO is critical for bacterial killing in the keratitis model.^44^

After infection with the clinical isolate, IL-1β and CXCL2, potent chemoattractants for neutrophils^45,46^ were both significantly reduced by GLY treatment. Unexpectedly, however, we found that CXCL2 was reduced almost to background levels when compared with IL-1β, particularly at 5 days PI. The observed reduction in CXCL2 expression is consistent with data from noninfectious inflammation studies, using a mouse model of cerulein-induced acute pancreatitis^47^ or in cerulein-stimulated pancreatic acinar cells.^48^ Reduction of IL-1β by GLY treatment also has been shown in a noninfectious mouse model of acute glaucoma^49^ and in rats with severe thermal injury.^50^

Treatment with GLY significantly reduced the neutrophil infiltrate in the infected cornea (MPO assay and histopathology) and these results are supported by Fakhari and coworkers^47^ who showed similar effects in cerulein-induced acute pancreatitis in mice. In addition, antibody neutralization of HMGB1^51^ in a murine model of cystic fibrosis, also led to reduction in neutrophils.

In addition to its anti-inflammatory effects, GLY treatment after infection with the clinical isolate also reduced the bacterial load (∼4.5 logs) at 5 days PI and reduced bacterial load after infection with the cytotoxic strain (∼2.5 logs) when compared with PBS controls. These data show a significantly better effect of GLY, when compared with thrombomodulin treatment, which reduced bacterial load by approximately 1 log^45^ at a similar time period after infection with the cytotoxic strain; the clinical isolate was not tested.

In this study we observed an apparent disconnect between reduced neutrophil infiltrate and yet a reduced bacterial load as described above. Therefore, we hypothesized that other mechanisms to control bacterial load are operative in this model. In the innate immune system, numerous effector molecules, including antimicrobial peptides (AMPs) such as defensins and cathelicidins play important roles in host defense.^52^ Both of the latter exhibit strong antimicrobial activity in various disease models.^53,54^ For example, in response to Pseudomonas keratitis, defensins (murine β defensin 2 and 3) are produced by the corneal epithelium and several cells in the corneal stroma, including neutrophils, macrophages, and fibroblasts.^55,56^ Silencing these defensins revealed their protective effects in a Pseudomonas keratitis model.^55,56^ Therefore, we examined the effects of GLY treatment on antimicrobial peptide production after infection with the noncytotoxic clinical isolate. Treatment significantly elevated murine beta defensin 2 (mBD2, encoded by gene *DEFB2*; beta defensin 2 in humans is encoded by the gene *DEFB4*) at all times tested, while CRAMP (human homologue, LL37), was elevated only at 3 days PI; mBD3 (human homologue, hBD2) was not changed. In mice^32^ and in a human mouse chimera^57^ infected with *P. aeruginosa*, it was shown that GLY treatment enhanced mBD1 and mBD3 and/or hBD1 production. In both of these studies,^32,57^ IL-10 and CCL2 were suppressed. Mechanistically, this was achieved by GLY binding to the glucocorticoid-like receptor and to HMGB1 and hence inhibiting production of IL-10 and CCL2. Although we did not see elevation of mBD3, nor test mBD1 after GLY treatment, we did observe reduction in IL-10 (mRNA), suggesting that a similar mechanism may be operative in the keratitis model.

Because of the success of the prophylactic treatment using GLY, we also tested whether treatment delayed by 6 hours PI would be effective. Although we did not observe significant reduction in clinical scores, opacity appeared reduced after GLY treatment. In addition, plate counts at 3 and 5 days PI were reduced significantly after GLY treatment, suggesting that there is great potential clinically for this agent in control of keratitis.

We also tested GLY in absorbance assays and found that the compound itself exhibited bacteriostatic/bactericidal activity against both bacterial strains. Others have tested various extracts of Glycyrrhiza glabra against both Gram negative and Gram positive bacteria, and found bactericidal effects that were concentration dependent, supportive of the data presented herein using GLY.^58^ These data raised the issue of the mechanism of GLY protection, suggesting that it is a bactericidal agent and that the anti-inflammatory effects are mediated by reduction of bacterial load not reduction of HMGB1. We suggest that this is not the case, as in the current work, we observed elevated, not decreased expression of AMPs, the former of which is consistent with a reduced bacterial load, and furthermore, many laboratories have reported that GLY is anti- inflammatory in sterile models of inflammation.^23,50,57,59,60^ For example, using a rat model of cerebral ischemia/reperfusion injury, Gong and coworkers^23^ revealed that GLY treatment significantly decreased the expression of proinflammatory cytokines TNF-α, iNOS, IL-1, and IL-6. Furthermore, they showed that this reduction is HMGB1 dependent as GLY treatment together with HMGB1 protein restored cytokine expression. Also, in an LPS-induced mouse mastitis model and in LPS-treated mouse mammary epithelial cells,^60^ GLY treatment significantly reduced MPO activity and protein expression of TNF-α, IL-1β, and IL-6. In addition, when we added rHMGB1 to GLY treatment, we found that plate count was significantly greater compared with GLY treated mice. Furthermore CBX also was capable of reducing load, but reduction was delayed until 5 days PI compared with GLY. The latter decreased load at 3 and 5 days PI and further, CBX did not reduce HMGB1. Collectively, these data suggest strongly that GLY has both bactericidal and anti-inflammatory functions and that HMGB1 levels are critical to these functions.

Treatment with GLY also was examined using a cytotoxic strain of the bacteria and was effective as it lowered clinical scores at 3 and 5 days PI and reduced opacity. Glycyrrhizin also was effective in reducing CXCL2 protein and the neutrophil infiltrate, but was less effective at reducing plate count (2.5 versus 4.5 logs when compared with the invasive strain).

We also found GLY treatment did not reduce HMGB1 levels after infection with the cytotoxic strain. This is consistent with differences in virulence factors between cytotoxic versus invasive strains.^61^ The former cause extensive necrosis, which would potentially release greater amounts of HMGB1 extracellularly than after infection with an invasive strain. This hypothesis is supported by the rHMGB1 data provided herein, where GLY treatment after infection with KEI 1025, the invasive strain, together with rHMGB1, abrogated GLY reduction of bacterial load in the cornea.

In summary, these data provide evidence that GLY treatment is effective in prophylactic/therapeutic treatment of bacterial keratitis with no adverse events. Its reduction of HMGB1 appears critical for optimum protection as rHMGB1 treatment with GLY reversed the latter's protective effect. Potential for further translational studies and for clinical applicability of GLY appears promising.
